# Impact of the COVID-19 pandemic on outcomes of acute ischemic stroke patients treated with endovascular therapy: A multicenter Canadian study

**DOI:** 10.1371/journal.pone.0316734

**Published:** 2025-02-10

**Authors:** Shenghua Zhu, Ammar Alam, Rebecca Thornhill, Vered Tsehmaister-Abitbul, Grant Stotts, Hailey Pettem, Richard Aviv, Ronit Agid, Aleksandra Pikula, Jai Jai Shiva Shankar, Genevieve Milot, Brian Van Adel, Samuel Yip, Manraj Heran, Robert Fahed, Fabio Settecase, Marlise P. dos Santos

**Affiliations:** 1 Department of Radiology, Radiation Oncology and Medical Physics and Department of Surgery, Section of Interventional Neuroradiology, The Ottawa Hospital, University of Ottawa, Ottawa, Ontario, Canada; 2 Department of Radiology, Division of Neuroradiology, Massachusetts General Hospital, Harvard Medical School, Boston, Massachusetts, United States of America; 3 Department of Medicine, Division of Neurology, University of Ottawa, Ottawa, Ontario, Canada; 4 Department of Medical Imaging, Division of Neuroradiology, Toronto Western Hospital, Toronto, Ontario, Canada; 5 Department of Medicine, Division of Neurology, University of Toronto, Toronto, Ontario, Canada; 6 Department of Radiology, University of Manitoba, Winnipeg, Manitoba, Canada; 7 Department of Surgery, Division of Neurosurgery, CHU de Québec - Université Laval, Quebec City, Quebec, Canada; 8 Divisions of Neurosurgery and Neurointerventional Surgery, McMaster University, Hamilton, Ontario, Canada; 9 Department of Medicine, Division of Neurology, University of British Columbia, Vancouver, British Columbia, Canada; 10 Department of Radiology, University of British Columbia, Vancouver, British Columbia, Canada; 11 University of Ottawa, Brain and Mind Research Institute, Ottawa Hospital Research Institute, Ottawa, Ontario, Canada; UCSF: University of California San Francisco, UNITED STATES OF AMERICA

## Abstract

**Objective:**

The novel coronavirus disease 2019 (COVID-19) pandemic led to the implementation of wide-ranging institutional infection control protocols. The purpose of this study is to determine the effect of the pandemic on outcomes of large vessel occlusion (LVO) acute ischemic stroke (AIS) patients treated with endovascular therapy (EVT).

**Materials and methods:**

Data were obtained from prospectively collected quality improvement stroke databases at six Canadian comprehensive stroke centres from March 11, 2020 to March 11, 2021. This patient cohort was compared to pre-pandemic patients consecutively treated with EVT from March 11, 2019 to March 10, 2020. The primary outcome is a 90-day modified Rankin Score (mRS). The secondary outcomes are angiographic time metrics.

**Results:**

A total of 1329 EVT patients (pre-pandemic n = 666) were included. The initial NIHSS was statistically significantly lower in the pandemic cohort. Other baseline patient characteristics were comparable between the two periods. Median (interquartile range, IQR) time from last seen normal (LSN) to emergency department (ED) (172 (68–316) vs 210 (97–382) min; p = 0.0001), LSN to puncture (235 (160–378) vs 280 (184–475); p < 0.0001), computed tomography (CT) to angiographic table (68 (44–108) vs 84 (57–125) min; p = 0.002), ED to angiographic table (65 (37–96) vs 80 (50–112) min; p = 0.001), CT to recanalization (117 (84–156) vs 130 (89–173) min; p = 0.038) and LSN to recanalization (279 (198–453) vs 327 (219–561) min; p = 0.002) were longer in the pandemic period as compared to the pre-pandemic. There were no significant differences in median time from angiographic table to arterial puncture (13 (8–19) vs 12 (9–16) min; p = 0.70) or arterial puncture to first pass (21 (14–31) vs 20 (14–30) min; p = 0.50). Patients were more likely to have favourable outcomes (mRS at 90 days score of ≤ 2) post-EVT pre-pandemic than pandemic (53% vs 44%; p = 0.02). Furthermore, analysis of the time interval from “LSN to arterial puncture” in relation to functional outcomes showed that the percentage of unfavorable outcomes increased among patients who underwent EVT within 240 minutes. Specifically, the rate of unfavorable outcomes rose from 32.9% to 42.9% (p = 0.37 for intervals under 150 minutes) and from 41.6% to 52.3% (p = 0.15 for intervals between 151 and 240 minutes) when comparing pre-pandemic to pandemic periods. However, the detrimental effect associated with the pandemic was diminished in patients who received EVT beyond 240 mins (p = 1.0).

**Conclusion:**

In this multicenter study involving six Canadian stroke centers, patients exhibited a higher probability of unfavorable long-term functional outcomes following EVT during the pandemic period compared to those in the pre-pandemic cohort, particularly during the first year of the pandemic.

## Introduction

The COVID-19 pandemic placed unprecedented strain on healthcare resources, significantly impacting the provision of timely care for acute conditions like stroke. The implementation of institutional infection protocols led to a shift of medical resources in the surgical field towards the medical care of patients with COVID-19 [1]. Furthermore, healthcare professionals have faced the challenges of maintaining their wellbeing and are constrained in giving timely care to those who require emergency and elective surgeries [2].

Stroke patient outcomes also worsened across the world. In the United States, stroke hospitalizations decreased by 22.3% in 2020 compared to 2019 [3]. Similarly, a study conducted in Catalonia found that individuals with COVID-19 and ischemic stroke had a more severe neurological deficit at admission, a lower proportion of favourable outcomes, and a significantly higher mortality rate [4].

Despite these international observations, there is a lack of nationwide data examining the impact of the COVID-19 pandemic on acute ischemic stroke (AIS) patients treated with endovascular therapy (EVT) in Canada. Our study aims to address this gap by providing a Canada-wide, multicenter analysis that is representative of most EVT cases in the country during this period.

In this study, we sought to understand the impact of COVID-19 on the outcomes of AIS patients treated with EVT across six Canadian stroke centers. We also measure angiographic time metrics and quality indicators to determine whether there were delays in thrombectomy treatment and reperfusion during the first year of the pandemic.

## Methods

**Fig 1:**
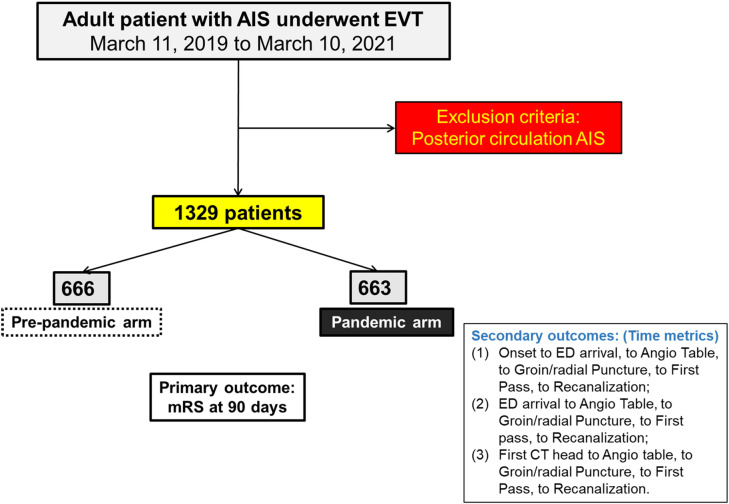
Flowchart of patient inclusion and exclusion criteria. This flowchart illustrates the selection process of patients included in the study from March 11, 2019, to March 11, 2021, across six Canadian stroke centers.

We conducted a retrospective multicentre cohort study at six Canadian stroke centres (university-affiliated tertiary care hospitals and all hospital names were deidentified with alphabetical letter from A to F). Data were extracted from prospectively collected quality improvement databases over two years from March 11, 2019 to March 11, 2021 at each participating site (Fig 1). We selected March 11, 2020, as the cutoff date to define the pre-pandemic and pandemic periods, corresponding to the WHO’s declaration of COVID-19 as a global pandemic [5]. This date marks the initiation of widespread public health measures and institutional protocol changes in Canada. Although the first confirmed COVID-19 case in Canada occurred on January 25, 2020, the prevalence remained low until after March 2020 [6]. Thus, the potential inclusion of COVID-19 patients in the pre-pandemic cohort is minimal. For the analysis, we used the following inclusion criteria: patient age 18 years and older; intracranial proximal arterial occlusion in the anterior circulation (intracranial carotid artery [ICA, ICA-T] or middle [M1/M2] or anterior [A1/A2] cerebral artery), demonstrated by computed tomography angiography (CTA), or digital subtraction angiography (DSA). Patients with AIS of the posterior circulation were excluded. The study was approved by the research ethics board at each site and a waiver of informed consent was obtained in accordance with institutional ethical guidelines and the data were accessed on July 26, 2023 for analyses.

### Clinical definitions

Endovascular treatment (EVT) refers to the endovascular retrieval of a blood clot within occluded brain arteries in acute ischemic stroke patients. The EVT modality for each patient was left to the discretion of the local interventionalists at each participating site. Stroke onset was defined as the time of symptom onset or the time that the patient was last seen normal if the onset of symptoms was unwitnessed. Alberta Stroke Program Early CT Score (ASPECTS) ranges from 0 to 10, with 1 point subtracted from 10 for any evidence of early ischemic changes in each defined region on non-contrast computed tomography (CT), with higher scores indicating a smaller infarct core [7]. Thrombolysis In Cerebral Infarction scale (TICI scale) is a grading system used to grade response to thrombolytic therapy in AIS [8,9]. Successful reperfusion was defined as a score on the TICI scale ≥ 2B [10].

### Outcome measures

In our analysis, the primary outcome was the functional status of AIS patients 90 days after EVT, measured using the Modified Rankin Scale (mRS). The mRS is a 7-point scale ranging from 0 (no symptoms) to 6 (death) [8]. The primary outcome is either dichotomized or trichotomized, based on mRS at 90 days. In dichotomization, the mRS score at 90 days of ≤ 2 points indicates a favourable outcome, while the mRS score of > 2 points indicates an unfavourable outcome. In trichotomization, mRS scores of 3–5 are unfavourable outcomes, and a score of 6 points is death. The secondary outcomes consisted of angiographic time metrics, including the time elapsed from Last Seen Normal (LSN) to emergency department (ED) arrival/angiographic table/arterial puncture/first pass/final recanalization (at/ap/fp/fr), first CT head to at/ap/fp/fr, and ED arrival to at/ap/fp/fr. Hemorrhages were evaluated following EVT procedures on follow up imaging.

The COVID-19 infection status of patients in both the pre-pandemic and pandemic cohorts was incomplete, as most patients’ COVID-19 status was not documented in the database. Due to this omission, we were unable to definitively determine which patients were COVID-19 positive or negative in either cohort. Despite this limitation, we included all patients who met the inclusion criteria to maintain the integrity of the baseline comparison and to evaluate real-world outcomes during this transitional period. This approach reflects the practical circumstances faced by healthcare providers during the early stages of the pandemic when testing was limited.

### Statistical analysis

Baseline characteristics of the study population are tabulated using descriptive statistics. Comparisons of medians or proportions between pre- vs. pandemic cohorts were performed using Mann-Whitney U test or Fisher’s exact test, respectively. For each angiographic time metric, a non-parametric two-factor ANOVA-type rank factorial test (“rankFD” in R https://cran.r-project.org/package=rankFD) was used to assess for potential interactions between pandemic effects and institution (stroke centre). For primary outcome analysis, a Fisher’s exact test was used to assess differences in dichotomous mRS90 between pre- and pandemic cohorts. For secondary outcome analysis, the LSN to arterial puncture interval was first trichotomized into three sub-intervals (<150, 151–240, > 240 minutes), and then Chi-squared tests were used to assess differences in dichotomous mRS at 90 days among the three sub-intervals. All statistical tests were two-tailed and a p-value below 0.05 was considered statistically significant. All analyses were performed with Rstudio (2022.07.2 + 576 “Spotted Wakerobin” Release, PBC, Boston, MA).

## Results

Summative analysis of six stroke centres included 1329 EVT patients (pre-pandemic n = 666). There were no significant differences between pre- and pandemic cohorts regarding age, sex, baseline mRS, or initial ASPECTS (Table 1). However, the initial NIHSS was significantly lower in the pandemic cohort (median (IQR) 17 (12–21) vs 15 (10–20), p < 0.001, Table 1).

**Table 1:** Baseline demographic characteristics among adult patients with acute ischemic stroke treated with EVT in six Canadian stroke centers.

Characteristic	Pre-pandemic	Pandemic	*p values*
Total patients included	666	663	
Female	328 (49.2%)	318 (48.0%)	0.532
Age (in years)			
Mean	71.1	70.1	
Median	73	72	0.26
Range	21–99	20–105	
Transfer from outside	266 (39.9%)	256 (38.6%)	0.637
Initial NIHSS			
Median (IQR)	17 (12–21)	15 (10–20)	*0.0004
Mean (SD)	16.1 (6.4)	14.8 (6)	
Initial ASPECTS			
Median (IQR)	8 (7–9)	8 (7–9)	0.09
Mean (SD)	8 (1.6)	7.8 (1.7)	
Baseline mRS			
Median (IQR)	0 (0–3)	1 (0–1)	0.677
Mean (SD)	1.4 (2)	1 (1.4)	
tPA (administered)	322 (48.3%)	292 (44.0%)	0.343
Post EVT death in hospital	40 (6%)	24 (3.6%)	0.213
General anesthesia	147 (22.1%)	92 (13.9%)	0.345
Stent required	178 (26.7%)	168 (25.3%)	0.247
LOS, mean (SD)	5 (8)	5 (8)	0.856
TICI 2b above	455 (68.3%)	480 (72.4%)	0.057

NIHSS, National Institutes of Health Stroke Scale; ASPECTS, Alberta Stroke Program Early CT Score; mRS, modified Rankin Scale; EVT, endovascular treatment; TICI, Thrombolysis in Cerebral Infarction scale; LOS, Length of stay. IQR, interquartile range; SD, standard deviation. *p < 0.05.

Several EVT time metrics were significantly longer in the -pandemic period as compared to the pre-pandemic: median (IQR) time from last seen normal (LSN) to ED (172 (68–316) vs 210 (97–382) min; p = 0.0001), LSN to puncture (235 (160–378) vs 280 (184–475); p < 0.0001), CT to angiographic table (68 (44–108) vs 84 (57–125) min; p = 0.002), ED to angiographic table (65 (37–96) vs 80 (50–112) min; p = 0.001), CT to recanalization (117 (84–156) vs 130 (89–173) min; p = 0.038) and LSN to recanalization (279(198-453) vs 327(219-561) min; p = 0.002). However, the pandemic median time from first pass to recanalization was slightly shorter (9(5-26) vs 7 (5–18) min; p = 0.046). There were no significant differences in median time from angiographic table to arterial puncture (13 (8–19) vs 12 (9–16) min; p = 0.70) or arterial puncture to first pass (21 (14–31) vs 20 (14–30) min; p = 0.50), as shown in Table 2.

**Table 2:** Summary of time intervals for patients with acute ischemic stroke treated with EVT, presented as medians with interquartile ranges (IQR).

Time intervals	Pre-pandemic		Pandemic		*p values*
	N	Median (IQR)	N	Median (IQR)	
Last seen normal to ED	625	172 (68–316)	613	210 (97–382)	*0.0001
Last seen normal to Angio	272	241 (149–421)	275	282 (175–443)	*0.0191
Last seen normal to Puncture	626	235 (160–378)	613	280 (184–475)	*< 0.0001
Last seen normal to First Pass	441	269 (190–425)	426	301 (211–518)	*0.0009
Last seen normal to Recanalization	341	279 (198–453)	317	327 (219–561)	*0.0023
CT to Angio	274	68 (44–108)	287	84 (57–125)	*0.0016
CT to Puncture	587	79 (49–133)	588	91 (57–144)	*0.0007
CT to First Pass	400	105 (72–165)	398	118 (78–178)	*0.0088
CT to Recanalization	340	117 (84–156)	323	130 (89–173)	*0.0379
ED to Angio	278	65 (37–96)	292	80 (50–112)	*0.0010
ED to Puncture	630	70 (41–105)	635	77 (44–109)	*0.1319
ED to First Pass	435	95 (68–134)	441	99 (68–137)	0.7265
ED to Recanalization	343	111 (80–155)	334	117 (77–155)	0.4919
Angio to Puncture	271	13 (8–19)	292	12 (9–16)	0.6981
Puncture to First Pass	437	21 (14–31)	443	20 (14–30)	0.5042
First Pass to Recanalization	234	9 (5–26)	228	7 (5–18)	*0.0457

*p < 0.05.

There was no observed interaction between institution and potential pandemic effects on the median time from LSN to ED (Institution p = 0.009; pandemic effect p = 0.0002; interaction p = 0.37), LSN to puncture (Institution p = 0.0001; pandemic effect p < 0.0001; interaction p = 0.62), ED to angiographic table (Institution p < 0.0001; pandemic effect p = 0.002; interaction p = 0.29), and LSN to recanalization (Institution p = 0.03; pandemic effect p = 0.005; interaction p = 0.63).

**Fig 2:**
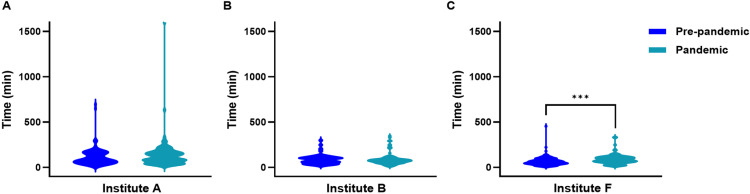
Violin plots showing the distribution of time intervals from CT to arrival at the angiography table for patients treated with EVT at (A) Institute A, (B) Institute B, and (C) Institute F. *** p < 0.0001.

There was a statistically significant interaction observed between the institution and pandemic effect on the median time from CT to angiographic table (Institution p < 0.0001; pandemic effect p = 0.008; interaction p = 0.03). However, the observed interaction with a larger institution effect is likely due to missing data from 3 centres, as shown in Fig 2.

**Fig 3:**
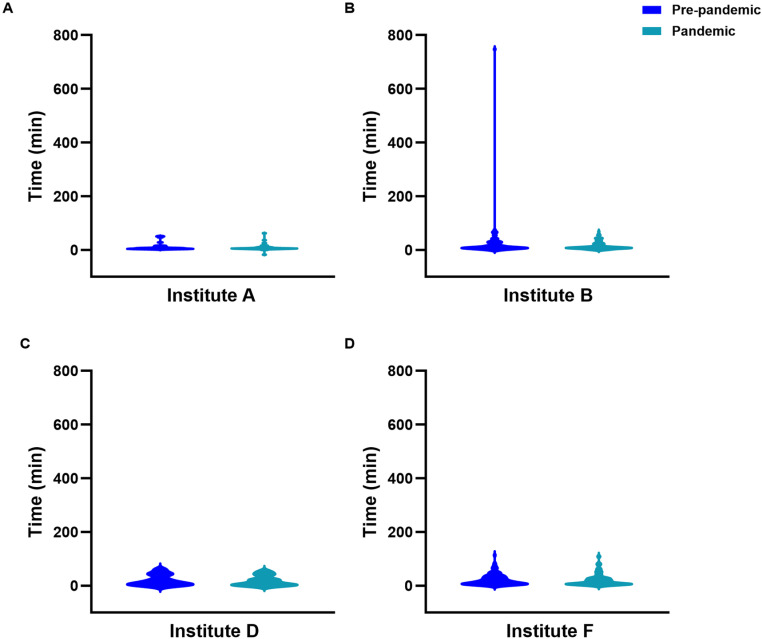
Violin plots showing the distribution of time intervals from first pass to recanalization for patients treated with EVT at (A) Institute A, (B) Institute B, (C) Institute D and (D) Institute F.

**Fig 4:**
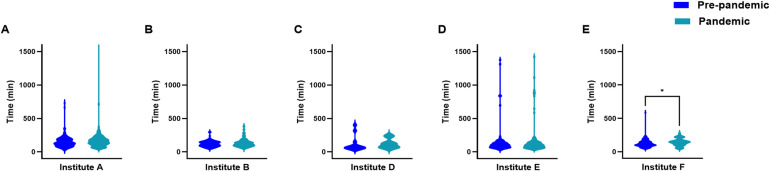
Violin plots showing the distribution of time intervals from CT to recanalization for patients treated with EVT at (A) Institute A, (B) Institute B, (C) Institute D, (D) Institute E and (E) Institute F. *  p < 0.05.

Despite there being no statistically significant interaction between institution and pandemic effects on median time from arterial puncture to first pass (Institution p < 0.0001; pandemic effect p = 0.18; interaction p = 0.94), first pass to recanalization (Institution p < 0.0001; pandemic effect p = 0.39; interaction p = 0.95), and CT to recanalization (Institution p < 0.0001; pandemic effect p = 0.04; interaction p = 0.7), the institution effect was the predominant factor in determining the observed median time difference for these intervals. Post-hoc analyses showed that Institute A had the shortest time from first pass to recanalization (Fig 3) while Institute E had the longest time from CT to recanalization (Fig 4).

For the primary outcome, Fisher’s exact test for dichotomous mRS90 demonstrated that 192 out of 363 patients had favourable outcomes in the pre-pandemic cohort compared to 139 out of 316 patients with favourable outcomes during the pandemic (53.5% vs 46.5%; p = 0.02). Similar proportion of favourable outcomes were observed in the trichotomized mRS groups using Chi-squared analysis (p = 0.002).

**Fig 5:**
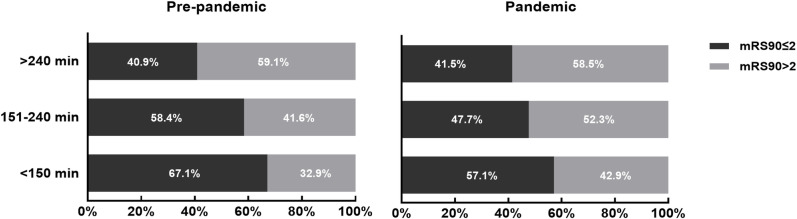
Distribution of dichotomous functional outcomes at 90 days by three time intervals from last seen normal to arterial puncture (<150 minutes, 151–240 minutes, > 240 minutes) in patients treated with EVT before and during the pandemic.

When the study population was further trichotomized according to LSN to arterial puncture time (<150 vs 151–240 vs > 240 minutes), the probability of favourable functional outcome declined with increased time interval from LSN to puncture time (Fig 5). However, the rate of unfavorable outcomes rose from 32.9% to 42.9% (p = 0.37 for intervals under 150 minutes) and from 41.6% to 52.3% (p = 0.15 for intervals between 151 and 240 minutes) when comparing pre-pandemic to pandemic periods. Interestingly, the detrimental effect associated with the pandemic was diminished in patients who received EVT beyond 240 mins (p = 1.0). The potential limitation from institutional effect was addressed with multiple Chi-squared analyses in 6 individual stroke centres which revealed statistically significant differences in 2 institutions.

Similar analyses were used to assess the association of the time from LSN to puncture with trichotomized functional outcomes between pre and pandemic followed by multiple Chi-squared analyses in 4 individual stroke centres. The rate of functional outcomes of mRS score > 2 and < 6 rose from 23.2% to 32.1% (p = 0.45 for intervals under 150 minutes) and from 24.8% to 39.8% (p = 0.02 for intervals between 151 and 240 minutes) when comparing pre-pandemic to pandemic periods (S1 Fig).

## Discussion

Multiple studies in the literature have reported the impact of COVID-19 on stroke and post-stroke recovery. Early studies conducted in Europe, the US, and China have reported both a decrease in stroke codes, and an increase in the severity of symptoms at presentation [11–14]. However, few studies have discussed either the outcome variables for patients presenting with AIS during the peak of the COVID-19 pandemic or the changing stroke characteristics during the initial COVID-19 surge recovery period. To our knowledge, this is the first Canada-wide study to assess the impact of the COVID-19 pandemic on long-term clinical outcomes in AIS patients treated with EVT.

Acute ischemic stroke is a highly time-dependent condition, and the rapid initiation of treatment remained vital during the pandemic. However, the fear patients faced and the challenges that stroke teams encountered when trying to balance the risk of infectious disease exposure and timely care presented hurdles worldwide. Time to EVT for AIS in current clinical practice is strongly associated with functional outcomes. Our data suggest that patients were more likely to have favourable outcomes (mRS at 90 days score of ≤2 points) post-EVT before the pandemic than during the pandemic. Indeed, the detrimental effect associated with the pandemic was diminished in patients who received EVT beyond 240 mins (p = 1.0). Whereas previous studies have reported mixed observations on short term functional outcomes post EVT in the early stage of the pandemic [15–17], the current paper advances our understanding by reporting on the long-term functional outcomes of patients treated with EVT during the first year of the pandemic. Interestingly, our data also demonstrated that the impacts of the pandemic on functional outcome were less pronounced as the time from symptom onset to EVT increased for patients.

Even though patients treated with EVT had lower baseline NIHSS during pandemic, there are still longer delays to presentation following the onset of symptoms during the pandemic period as noticed in prior series [12,18]. We also found there was significant delay in transporting patients from the emergency department to the angiography suite than in the pre-pandemic phase. This prolongation might be explained by more extensive (COVID-19) work-up before an EVT procedure during the pandemic. As a result, we have conducted a separated evaluation of the variations in safety protocols across the six stroke institutions which is not the primary focus of this manuscript. We found the observed prolongation is highly likely to be attributable to the implementation of new safety protocols by all institutions during the pandemic. For instance, pre-procedural intubation was mandated in certain institutes during the early phase of the pandemic prior to EVT.

Interestingly, despite a significant delay in the workflows after introduction of the new safety protocols, the mean time from angiographic table to arterial puncture and arterial puncture to first pass remained unchanged from the previous year pre-pandemic. This could be attributed to less severe stroke in patients during the pandemic as evidenced by the lower baseline NIHSS and slightly shorter time required from first pass to recanalization in this cohort. Furthermore, it could be also argued that patients with less comorbidities and lower risk of complications were preferentially selected to receive mechanical thrombectomy during the pandemic, which unfortunately, remains speculative, due to the lack of the information collected regarding underlying comorbidities in the current study. More importantly, this observation highlights that the pandemic safety protocols adopted in the angiography suites at the six Canadian major stroke institutes seeking to increase safety for healthcare teams did not adversely affect procedural time metrics.

Our study demonstrates the initial NIHSS at the time of initial hospital assessment was significantly lower among patients who presented during the early phase of the pandemic, which may imply that these patients presented with less severe stroke symptoms than the pre-pandemic cohort. However, the lower NIHSS observed at admission for pandemic patients appears counterintuitive, particularly given the stay home order that was issued at the beginning of the pandemic. This observation also contradicts previously published studies in which patients presented with more severe stroke symptoms during the first few months of the pandemic [19]. The lower NIHSS during the early pandemic seen in our study is likely the result of a combination of factors related to health system dynamics, patient behaviors, and even changes in stroke presentations, each of which could reflect regional or national differences in pandemic response. Understanding the underlying multifactorial bases for these differences could potentially help healthcare systems adapt and improve stroke care during ongoing and future public health crises.

The major strength of our study lies in the nation-wide comprehensive data collection on all consecutive AIS patients treated with EVT during the first year of the SARS CoV2 pandemic across six Canadian stroke centers with documented long-term outcomes. As noted in previous studies, the epidemiology and impact of COVID-19 vary significantly across different regional and international cohorts. Our multi-center study mitigates local heterogeneities across various provincial jurisdictions, thereby offering a more generalizable understanding of the early impact of the pandemic in Canada.

## Limitations

Our study has several limitations that should be acknowledged. A significant limitation of our study is the incomplete data on the COVID-19 infection status of patients in both cohorts. The absence of comprehensive COVID-19 testing data prevents us from identifying and excluding COVID-19 positive patients from the pre-pandemic cohort and limits our ability to perform subgroup analyses based on infection status during the pandemic period. This limitation could potentially introduce confounding effects, as COVID-19 infection itself may influence stroke presentation, severity, treatment response, and outcomes [4].

Another limitation of our study was the lack of detailed data on comorbidities and variations in care availability. Comorbidities such as hypertension and diabetes have been shown to significantly worsen stroke outcomes, affecting both severity and recovery trajectories [20]. Furthermore, studies have reported that the COVID-19 pandemic disrupted access to essential rehabilitation services, leading to poorer functional outcomes for stroke patients [21]. Our inability to adjust the outcome for these comorbidities and variations in care availability may affect the interpretation of our findings.

In addition, a selection bias in the identification of possible EVT candidates cannot be excluded, as the criteria for selecting patients with large vessel occlusion to the angiography suite in the pandemic cohort remain unknown. However, the impact of this selection bias is likely minimal and is most likely mitigated by the multicenter nature of the analysis. Finally, our multi-center study was meant to alleviate the regional heterogeneities among different institutions. However, we did notice that there were statistically significant institutional effects when assessing the relationship between the time interval “LSN to arterial puncture” and functional outcomes. These findings may be related to underlying variations in the new stroke protocols implemented during the pandemic. Although the majority of stroke centers worldwide adapted their protocols based on national and/or international (WHO) guidelines, further investigation of inter-institutional differences is warranted. Such an investigation may provide valuable insights into future public health crises beyond stroke care within acute medicine.

## Conclusion

In this multicenter Canadian study, AIS patients treated with EVT during the first year of pandemic experienced worse long-term functional outcomes compared to the pre-pandemic cohort, emphasizing the impact of pandemic-related delays on stroke care. Future research should focus on developing resilient stroke care models to ensure timely access to care for stroke patients during ongoing and future pandemics.

## Supporting information

S1 Fig Distribution of trichotomized functional outcomes at 90 days by three time intervals from last seen normal to arterial puncture (<150 minutes, 151–240 minutes, > 240 minutes) in patients treated with EVT before and during the pandemic. (TIF)

S1 File Raw dataset from six Canadian stroke centers, deidentified with letters A–F in the first column. The second column indicates pre-pandemic (0) versus pandemic (1) status. Additional columns contain baseline demographic information (e.g., patient age), initial clinical scores (NIHSS and ASPECTS), angiographic time metrics, length of hospital stay, and functional outcomes (mRS at 90 days).
(XLSX)

## Abbreviations

COVID-19: Coronavirus Disease 2019
LVO: large vessel occlusion
AIS: acute ischemic stroke
EVT: endovascular therapy
ASPECTS: Alberta Stroke Program Early CT Score
