# Supplementary figures and images for: Impact of the COVID-19 pandemic on outcomes of acute ischemic stroke patients treated with endovascular therapy: A multicenter Canadian study
Source: PLoS One. 2025 Feb 10;20(2):e0316734. doi: 10.1371/journal.pone.0316734 (PMC11809926; doi:10.1371/journal.pone.0316734)

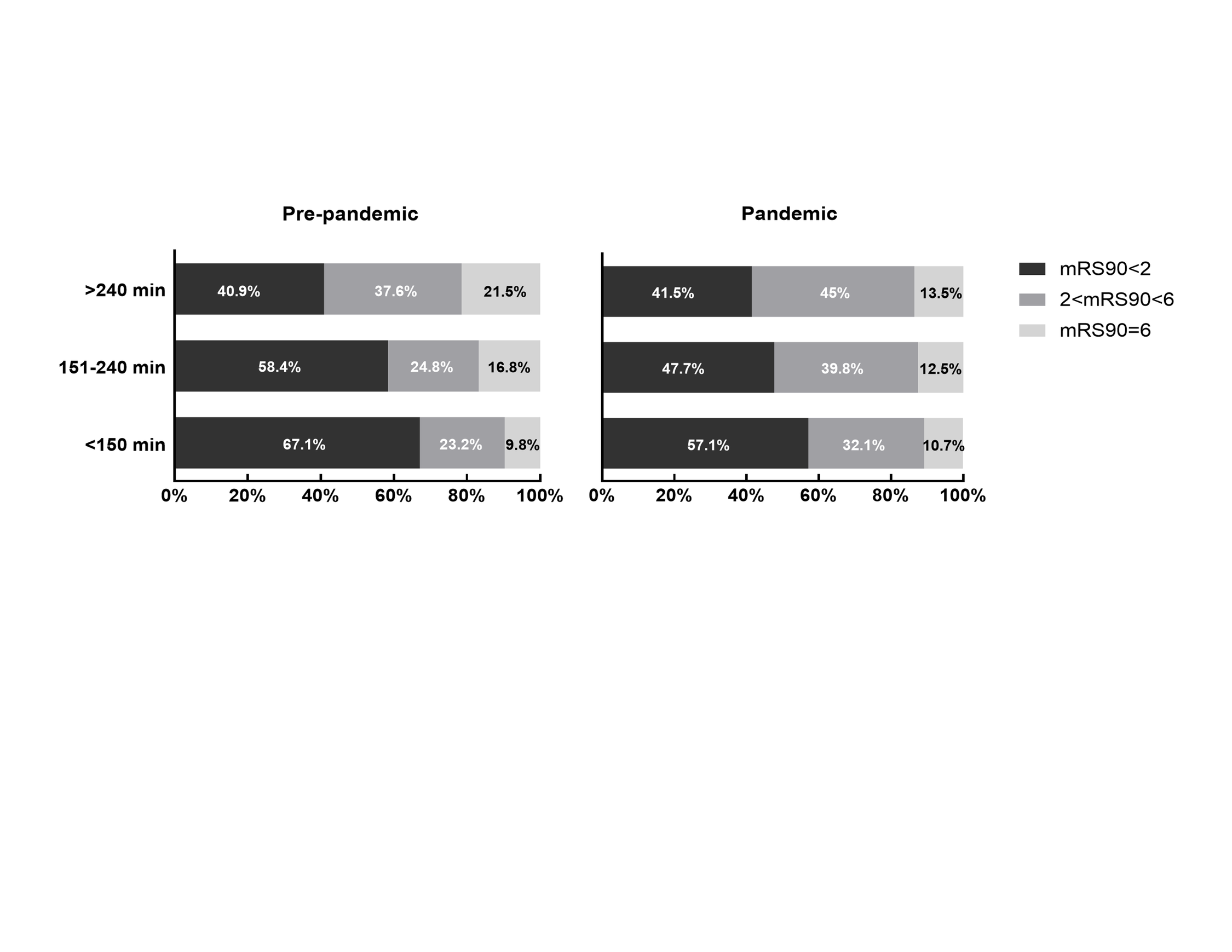

Supplement: S1 Fig — (TIF) [file pone.0316734.s001.tif]
